# The Diabetes Remission Clinical Trial (DiRECT): protocol for a cluster randomised trial

**DOI:** 10.1186/s12875-016-0406-2

**Published:** 2016-02-16

**Authors:** Wilma S. Leslie, Ian Ford, Naveed Sattar, Kieren G. Hollingsworth, Ashley Adamson, Falko F. Sniehotta, Louise McCombie, Naomi Brosnahan, Hazel Ross, John C. Mathers, Carl Peters, George Thom, Alison Barnes, Sharon Kean, Yvonne McIlvenna, Angela Rodrigues, Lucia Rehackova, Sviatlana Zhyzhneuskaya, Roy Taylor, Mike E. J. Lean

**Affiliations:** University of Glasgow, University Avenue, Glasgow, G12 8QQ UK; Newcastle University, Newcastle upon Tyne, Tyne and Wear NE1 7RU UK; Counterweight Ltd, Edinburgh, UK

**Keywords:** Type 2 diabetes, Weight management, Total diet replacement, Primary care

## Abstract

**Background:**

Despite improving evidence-based practice following clinical guidelines to optimise drug therapy, Type 2 diabetes (T2DM) still exerts a devastating toll from vascular complications and premature death. Biochemical remission of T2DM has been demonstrated with weight loss around 15kg following bariatric surgery and in several small studies of non-surgical energy-restriction treatments. The non-surgical Counterweight-Plus programme, running in Primary Care where obesity and T2DM are routinely managed, produces >15 kg weight loss in 33 % of all enrolled patients. The Diabetes UK-funded Counterpoint study suggested that this should be sufficient to reverse T2DM by removing ectopic fat in liver and pancreas, restoring first-phase insulin secretion.

The Diabetes Remission Clinical Trial (DiRECT) was designed to determine whether a structured, intensive, weight management programme, delivered in a routine Primary Care setting, is a viable treatment for achieving durable normoglycaemia. Other aims are to understand the mechanistic basis of remission and to identify psychological predictors of response.

**Methods/Design:**

Cluster-randomised design with GP practice as the unit of randomisation: 280 participants from around 30 practices in Scotland and England will be allocated *either* to continue usual guideline-based care or to add the Counterweight-Plus weight management programme, which includes primary care nurse or dietitian delivery of 12-20weeks low calorie diet replacement, food reintroduction, and long-term weight loss maintenance. Main inclusion criteria: men and women aged 20-65years, all ethnicities, T2DM 0-6years duration, BMI 27-45 kg/m^2^. Tyneside participants will undergo Magnetic Resonance (MR) studies of pancreatic and hepatic fat, and metabolic studies to determine mechanisms underlying T2DM remission. Co-primary endpoints: weight reduction ≥ 15 kg and HbA1c <48 mmol/mol at one year. Further follow-up at 2 years.

**Discussion:**

This study will establish whether a structured weight management programme, delivered in Primary Care by practice nurses or dietitians, is a viable treatment to achieve T2DM remission. Results, available from 2018 onwards, will inform future service strategy.

**Trial registration:**

Current Controlled Trials ISRCTN03267836. Date of Registration 20/12/2013

**Electronic supplementary material:**

The online version of this article (doi:10.1186/s12875-016-0406-2) contains supplementary material, which is available to authorized users.

## Background

T2DM is closely linked to obesity, particularly adult weight gain, and is the main contributor to rising healthcare costs of obesity [1, 2]. Whilst it seldom develops with BMI <21 kg/m^2^, most people with T2DM have a BMI >25 kg/m^2^ and around 50 % have a BMI >30 kg/m [2–4]. With BMI >35 kg/m^2^, 20 % of all men and 11 % of women have known diabetes [5].

There is consistent evidence that modest sustained weight loss, using a variety of diet and lifestyle approaches to achieve 5–10 % weight loss, will prevent the onset of most new cases of T2DM in those with pre-diabetes [6, 7]^,^, and that it improves *all* aspects of diabetes control (glycaemia, blood pressure, lipids and microvascular damage [8] with reductions in drug doses^,^[9, 10]. Advice to lose and maintain 5–10 % weight loss, by diet and exercise is included in most clinical guidelines [11, 12]. However, most T2DM patients in the UK are now managed in primary care, many do not receive the necessary specialist lifestyle advice and support, from trained staff, to achieve 5 % weight loss. Financial incentives are provided only for diagnosis and for prescribing anti-diabetic drugs [13].

Recognising changes in obesity prevalence, and also recent evidence for the benefits of more aggressive interventions including surgery, the 2010 SIGN Obesity guideline set a new weight loss/ maintenance target of >15–20 % for those with BMI >35 kg/m, or >30 kg/m^2^ with serious medical complications such as T2DM [11]. This degree of weight loss most reliably led to diabetes remission in a randomised trial of bariatric surgery [14] and several studies have suggested that similar weight loss is necessary to normalize blood glucose and insulin [15, 16]. There is also indirect evidence that such weight loss might normalise life expectancy in those with T2DM [4]. However, routine NHS diabetes care seldom aims for weight loss of >15 kg (commonly equivalent on average to >15 %: most studies have average weight close to 100 kg in obese individuals with T2DM) and vanishingly few obese people with T2DM achieve this goal.

Bariatric surgery can reverse the metabolic abnormalities of T2DM, at least in patients up to 6 years after diagnosis [17] and the proportion of individuals achieving normal blood glucose is determined primarily, by the extent of weight loss [18]. However the Counterpoint study [16] demonstrated that a broadly similar benefit can be reproduced by negative energy balance alone. Eleven people with T2DM (nine male and two female, BMI 33.6 ± 1.2 kg/m2) were studied before and after 1, 4 and 8 weeks of a 2.5 MJ (600 kcal)/day liquid diet. Results showed a mean weight loss of 15.3 kg and a rapid return of fasting blood glucose to normal, which persisted for up to 3 months after return to normal diet. The underlying changes in liver and pancreas fat were consistent with the Twin Cycle Hypothesis [19]. Publication of the Counterpoint study, demonstrating remission of T2DM and normalization of first-phase insulin response following weight loss of about 15 kg, prompted a massive response from people with T2DM who wanted to try to reverse their disease by weight loss. Evaluation of 77 reported experiences of self-directed weight loss indicated diabetes remission in 61 % overall: 80 % with >20 kg weight loss; 63 % with 10-20 kg weight loss; and 53 % with <10 kg weight loss [20]. These data, while self-reported and from self-selected motivated patients, indicate both a desire amongst those with T2DM to reverse their diagnosis, and that well-maintained weight loss leading to sustained remission of T2DM is achievable in a community setting.

The feasibility of a non-surgical approach for weight management has been demonstrated within routine NHS primary care. That feasibility study, mainly in non-diabetic patients (*n* = 91, mean BMI >48 kg/m^2)^ found that an 810 kcal/d Total Diet Replacement (TDR), followed by structured food reintroduction and then a long-term weight-maintenance programme, was acceptable to both staff and patients. This programme resulted in a mean loss of about 17 kg in 12 weeks [21]. At 12 months, at least 30 (33 %) of the 91 enrolled patients remained >15 kg lighter than at baseline. These observations indicate that it is possible to achieve, and maintain, substantial weight loss for many individuals by non-surgical methods. A recent systematic review of controlled studies evaluating very low energy diets (800 kcal/d), in individuals diagnosed with T2DM, found that these interventions led to greater weight losses at 3–6 months than minimal intervention or standard care [22]. However a longer and larger study is needed to determine whether i) this is a viable treatment for putting T2DM into remission, and ii) this approach is potentially transferable to a larger scale as part of routine GP care. The latter is particularly important because most overweight people with T2DM in the UK are managed in primary care rather than in specialist hospital clinics.

It is also important to extend our understanding of the mechanisms, which underlie the development of T2DM in susceptible individuals, and those responsible for achieving and maintaining remission. Over an eight-week study period, the Counterpoint study [16] showed a return to near-normal first-phase insulin secretion, concurrent with a loss of abnormal ectopic fat accumulation in liver and pancreas. The subsequent Counterbalance study [23] found that this dramatic reversal of T2DM occurs most reliably in patients with diabetes duration under 6 years since diagnosis. There is always some uncertainty about the duration of a diabetic state prior to diagnosis, but with known duration over 8 years since diagnosis, remission was much less likely.

### **Study Objectives:** (as set out in application & Ethics submission)

- To determine whether a programme designed to achieve remission of T2DM to normal glucose tolerance by substantial weight loss can be effectively delivered, within the routine Primary Care setting where T2DM is normally managed
- To evaluate potential prospective metabolic and psychological markers for a good response to the proposed treatment programme
- To define the mechanisms of long-term remission of diabetes, in particular examining the relationships of VLDL-triglyceride production rates to both pancreas fat content and beta cell function.
- To evaluate the barriers to successful reversal of T2DM at both the individual and service level

Data will be collected for possible future health economics analyses

**Co-primary endpoints**:

- Reduction in weight of 15 kg (assumed equal on average to 15 %) or more at one year;
- Remission of diabetes (HbA1c <48 mmol/mol) at 1 year.

**Secondary endpoints**:

- Quality of life
- Physical Activity
- Serum Lipids*,* Liver function tests, Urea & Electrolytes, plasma glucose
- Programme acceptability

## Methods/Design

The study will use a cluster-randomised controlled design, with GP practice the unit of randomisation. Randomisation at a practice level will avoid contamination between groups and allow consistent advice from practice nurses/dietitians.

Randomisation of practices will be conducted centrally and independently of the research team, by the Robertson Centre for Bio-Statistics, University of Glasgow. Practices will be allocated using a minimisation method to maintain the required balance across intervention groups within each study region (Scotland or England) and practice list size (>5700 or ≤ 5700).

Funding was awarded by Diabetes UK following extensive peer review. Ethical approval was obtained from the West of Scotland Research Ethics Committee (reference number: 13/WS/0314).

### **Recruitment**

#### *General practices*

Recruitment of general practices will be undertaken in multiple Health Board areas in Scotland and in the Newcastle -upon-Tyne NHS Foundation Trust area in the North East of England. Practices will be invited to participate by the Primary Care Research Network (PCRN). Practices expressing interest in participation will be contacted by the research team and recruited to the study.

#### *Participant recruitment*

**Fig. 1:**
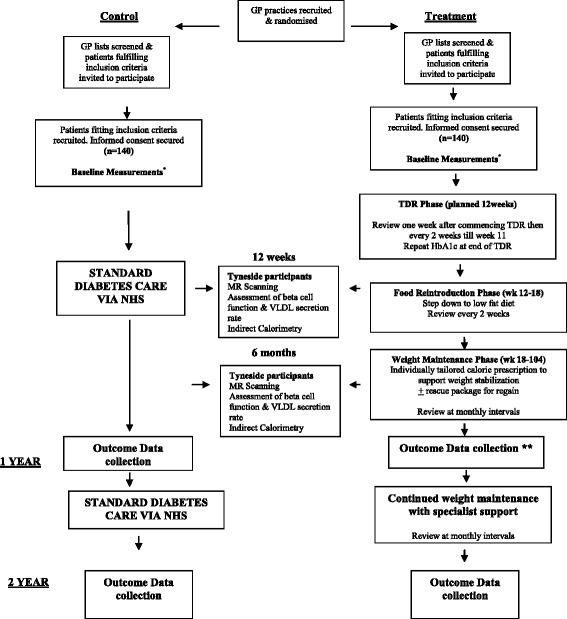
Study flow chart

Two hundred and eighty participants (140 per arm) will be recruited to the study from general practice (Fig. 1). Potential participants will be identified by a computerised search of GP records, undertaken by staff from PCRN. Lists generated by the search will be reviewed by GP’s to remove individuals likely to be ineligible, or unsuitable to approach because of co-morbidity or other practical or medical obstacles to participation identified by the GP. Reasons for exclusions will be recorded. Potential participants will be sent an invitation to participate on behalf of their GP. The invitation includes a written information sheet providing details of the treatment arm to which their GP practice is randomised. Patients are asked to indicate whether, or not, they are interested in participating, using reply-paid envelopes. Applying adaptive trial principles in response to early process evaluation which revealed low uptake by eligible patients, under substantial amendments the invitation letter was re-formatted as a coloured leaflet. A single reminder letter will be posted, after one month, to patients who do not respond to the initial invitation. Telephone calls will be made to those patients who are eligible and to those who had previously joined a register to indicate their willingness to be approached for clinical trials. Posters in primary care waiting rooms will also remind patients, and encourage those interested to respond if they had received an invitation.

Patients who respond as interested in participating will be contacted by a member of the research team and invited to attend for a screening appointment where the study will be fully explained and discussed. Informed consent will be secured from all participants, for 2-years on the trial protocol and for indefinite long-term data collection from their medical records and through national records linkages.

#### *Inclusion criteria*

- Written informed consent
- Men and women aged 20–65 years
- T2DM of duration 0–6 years (diagnosis based on 2 recorded diagnostic-level tests, HbA1c and/or blood glucose)
- HbA1c ≥ 48 mmol/mol at the last routine clinical check, within last 12 months if on diet alone
- HbA1c ≥ 43 mmol/mol if on treatment with oral hypoglycaemic agents
- Body Mass Index (BMI) >27 kg/m^2^ and <45 kg/m^2^

#### *Exclusion criteria*

- Current insulin use
- Recent routine HbA1c ≥108 mmol/mol
- Weight loss of >5 kg within the last 6 months
- Recent eGFR <30 mls/min/1.73 m^2^
- Substance abuse
- Known cancer
- Myocardial infarction within previous 6 months
- Severe heart failure defined as equivalent to the New York Heart Association grade 3 (NYHA)
- Learning difficulties
- Current treatment with anti-obesity drugs
- Diagnosed eating disorder or purging
- Pregnant/ considering pregnancy
- Patients who have required hospitalisation for depression or are on antipsychotic drugs
- People currently participating in another clinical research trial
- People with contraindications for MR scanning

### **Trial procedures**

Practices randomised to control will continue to deliver usual diabetes and obesity management as per current clinical guidelines. Detailed information on usual care pathways will be recorded in each area. Participants recruited in these practices will be followed up for 2 years and seen on 3 occasions by the study research dietitians, for study outcome data collection (Additional file 1: Table 1).

Practices randomised to intervention will continue usual guideline-based care and also deliver Counterweight Plus, which includes a Total Diet Replacement (TDR) phase followed by structured food reintroduction (FR) and a structured support programme for long term weight loss maintenance. The intervention will be delivered to each participant individually, at their general practice, by either a practice nurse or local dietitian, depending on practice preference and availability of dietetic support. Participants will attend for 35 appointments over the 2-year intervention period (Additional file 1: Table 1).

Training (8 h) for the practice nurses/dietitians in delivery of Counterweight Plus and study protocol will be provided by the study research dietitians who are trained in the package developed for, and improved following, the Counterweight feasibility study in primary care [21]. A detailed description of the practical dietetic input will be published separately.

### **TDR phase (0-12wks)**

A commercial micronutrient-replete 825–853 kcal/d liquid formula diet (soups and shakes) will be provided (Cambridge Weight Plan) to replace usual foods, with ample fluids (2.25 L), for 12 weeks. Oral hypoglycaemic agents (OHA), antihypertensive and diuretic drugs will be withdrawn on commencement of TDR, and reintroduced (as per study protocols) if T2DM or hypertension returns. Aspirin will be continued if prescribed because of a previous MI (prior to the previous 6 months), but discontinued if prescribed solely because of T2DM. Beta-blockers prescribed for the management of angina will be continued. A soluble fibre supplement (Fybogel 2 × 3.5 g/day) will be prescribed to reduce constipation.

Participants will return for review one week after commencement on the TDR and at 2 weekly intervals thereafter until the commencement of the FR stage (Fig. 1).

To allow some flexibility for patients whose commitments, or life events, prevent achievement of 15 kg at 12 weeks, or if individuals wish to achieve more weight loss, the TDR phase may continue up to 20 weeks. If BMI falls below 23 kg/m^2^ during the TDR phase, participants will be moved forward to the FR and weight loss maintenance phases.

### **Food reintroduction phase (weeks 12–18)**

The FR phase includes a stepped transition to a food-based diet based on the “Eatwell” guidelines [24] while reducing TDR. To allow flexibility for participants whose confidence varies, the FR phase can be varied between protocol-defined limits of 2–8 weeks before switching to full food-based weight loss maintenance. Participants will monitor weight on a weekly basis and compare this with caloric intake and activity levels. Participants will return for review at 2 weekly intervals throughout the FR phase.

### **Weight loss maintenance phase (weeks 19–104)**

Participants will be advised to follow a food based diet and will be provided with an individually tailored energy prescription, to support weight stabilisation and prevent weight regain. The option of using one sachet of formula diet per day for the duration of weight loss maintenance will also be available to participants. Review appointments will be at monthly intervals.

All participants allocated to the intervention will be provided with printed support materials describing the management plan and support for each phase of the intervention. Those who are physically capable will be advised to increase daily physical activity. Step-counters will be provided with the recommendation to aim to reach and maintain their individual sustainable maximum, up to 15,000 steps/day [25, 26].

### **Relapse management for weight regain or re-emergence of diabetes**

If weight regain occurs, or if diabetes is found to have returned (HbA1c ≥48 mmol/mol) at any time during the 18-month weight loss maintenance stage, ‘rescue plans’ to reverse weight gain will be offered.

1. Weight regain of >2 kg: offer the use of TDR to replace one or two main-meal per day for 4 weeks, offer orlistat 120 mg tid, with each meal.
2. Weight gain of >4 kg, or to <15 kg below starting weight or if diabetes recurs: offer 4 weeks TDR with fortnightly practice nurse/dietitian review and then a 2–4 week FR (as described above). Individualised dietary advice, based on the Eatwell guidelines [24], and physical activity targets will be reinforced for weight loss maintenance. Orlistat treatment, as above, will be offered for the remainder of the weight loss maintenance period, with repeat advice to restrict dietary fat.

Relapse management will include an exploration of the reasons for weight regain, and anticipatory support to prevent recurrence.

#### **Patient safety**

Intervention participants will be closely monitored throughout the study (Additional file 1: Table 1). Blood pressure, postural symptoms and capillary blood glucose will be monitored at each appointment. Anti-hypertensive therapy or oral hypoglycaemic agents will be reintroduced if necessary according to a defined proforma (Appendix 1: Protocols for management of blood pressure & inadequate glucose control). All observations/results which may pose a risk to health will be discussed with the participant and their GP. Enquiry at each study visit will identify the occurrence of adverse events which will be recorded.

#### **Protocol delivery fidelity**

A standard training protocol has been developed to minimise variability and maintain intervention protocol fidelity across all practices. Practitioner mentoring (nurse or dietitian) will be carried out, by the study research dietitians, during each stage of the intervention, and feedback provided to practitioners as required. Variability in primary outcome assessments (body weight, T2DM status) will be minimised by using calibrated equipment and quality-controlled assays of blood glucose and HbA1c.

### **Measurements**

The measurements taken at each stage of the DiRECT study are detailed in Additional file 1: Table 1.

Height will be measured to the nearest mm, with the Frankfort plane horizontal, using a portable stadiometer (Chasmors Ltd, London). Body weight will be measured to the nearest 100 g in light clothing without shoes using Class 111 approved calibrated scales (Marsden Group UK). Waist circumference will be measured halfway between the point of the lowest rib and the iliac crest. Hip circumference will be measured at the maximum circumference around the buttocks. Blood pressure will be measured with patients seated, at rest, with legs uncrossed for at least 5 min.

Blood will be collected to measure: serum urea and electrolytes, liver function tests, serum total cholesterol, HDL cholesterol and triglycerides, HbA1c, plasma glucose, metabolomic profiling and other novel metabolic/protein markers. Urine will be collect for: assessment of microalbuminuria and for proteomics.

#### *Objectively measured health behaviours*

Physical activity will be assessed in all participants at baseline, 12 and 24 month follow-up, using GENEActiv accelerometers, a fully waterproof wrist-worn tri-axial, raw data accelerometer, for activity and sleep tracking in free living studies (http://www.geneactiv.org/actigraphy/geneactiv-original). The device will be worn by participants for 9 days at each data collection time point.

#### *Mechanistic studies (Tyneside)*

Tyneside participants (intervention (68); control (20)) will participate in mechanistic studies to define the physiological mechanisms underlying the long term remission of T2DM (Additional file 1: Table 1). Liver and pancreas fat will be quantified using 3-point Dixon MR scanning [16]. VLDL1 secretion rates will be measured by plasma accumulation during inhibition of clearance [27]. Beta-cell function will be assessed using the stepped insulin secretion test with arginine [16]. Indirect calorimetry, using a Quark RMR indirect calorimeter (COSMED, Rome, Italy), will estimate rates of whole body lipid oxidation as part of the assessment of lipid dynamics. Whole body glucose oxidation will also be measured [28].

#### **Programme acceptability, process evaluation and Quality of Life**

##### Participants

At least 10 intervention and 10 control participants in both Scotland and Tyneside will be invited to participate in semi-structured face-to-face or telephone interviews focusing on experiences, barriers, facilitators and behaviour strategies for self-regulation. The exact number of patients recruited will follow standard guidelines for data saturation in qualitative research [29]. Intervention participants will be interviewed on four occasions during the TDR phase using semi-structured theory domain [30] interviews, before/at the commencement of TDR, 6–8 weeks afterwards, and at months 1 and 12 of the weight maintenance phase. Control group participants will be interviewed twice, at the commencement (parallel to TDR phase) and at the end of their participation in the study (parallel to month 12 of the weight maintenance phase). Interviews will be carried out at the participant’s GP practice, at the University premises, or by telephone.

Secondary trial outcomes for Quality of Life (EQ-5D-3 L) will be measured at baseline, 12 and 24 months. Psychological process measures (intention, perceived control and satisfaction with treatment) will be quantified using questionnaires developed specifically for the study in both intervention and control groups. Questionnaires will be administered, by the study research dietitians, at baseline and 12 months for all participants. Intervention participants will complete an additional questionnaire at the end of the TDR period.

#### *Ecological Momentary Assessment (EMA)*

All interviewed participants (20 in intervention group and 20 in control group) will be invited to complete an Ecological Momentary Assessment (EMA) for 12 weeks from the baseline interview and for 8 weeks (weeks 3–10 inclusive) during the weight maintenance phase.

EMA will prompt participants to record experiences by responding to a set of daily questions measuring adherence to the dietary prescription as well as other measures associated with adherence. For participants who do not wish to complete the EMA via mobile phone, procedures will be provided for on-line or paper- and-pencil-based completion.

#### *Healthcare professionals*

A purposive convenience sample of 10–15 health care professionals (nurses & dietitians) involved in delivering the intervention will be invited for interview and the barriers and facilitators to implementation, and ease of engagement with the intervention, will be evaluated using semi-structured theory domain interviews. Standard guidelines for qualitative research will be used to achieve data saturation [29]. Interviews will be conducted either face-to-face at the health professionals place of work, or by telephone if preferred by the health professional.

### **Statistical analysis**

Primary outcome measures will be analysed in a hierarchical fashion, first analysing reduction in weight of 15 kg or more as a binary outcome with significance assessed at the 5 % significance level, followed, if the first test is significant, by test of remission of diabetes status as a binary outcome also at the 5 % level of significance. The overall type I error will not exceed 5 % because of the hierarchical nature of the testing.

Data will be analysed initially on an intention-to-treat (ITT) basis at the 12 month time point. For participants who discontinue the formal weight management programme the follow-up weights (from routine attendances) and the end of study diabetes status will be used. Secondary analyses will include fasting blood glucose at 6 months in those maintaining weight loss of 15 kg or more; a secondary per protocol ‘completer’s analyses’ of those who complete the intended management (irrespective of their dietary adherence or weight -change); analysis of BP and blood lipid outcomes; mechanistic parameters analysed according to blood glucose outcomes. Participants reporting drug intolerance, diet intolerance or poor-compliance will be recorded and these participants will be included in the ITT analysis.

#### *Non-responders*

Anonymised data (age, sex, BMI and duration of diabetes) for all patients identified as satisfying the recruitment criteria and invited to participate will be recorded. Data for non-responders will be compared with the same data from those who agree to participate in the study. This will permit identification of any recruitment bias, determine the representativeness of study participants, and help establish generalisability of the trial findings.

#### *Participant withdrawal*

Participants who withdraw from the intervention protocol, or who fail to return for follow-up assessments, will continue to have data collected from their routine diabetic clinic/GP visits, unless they specifically withdraw consent for this. Data analysis will use best available follow-up weights (closest within a window of ±3 months from routine attendances) and end of study diabetes status for participants who discontinue the formal weight management programme. Drug intolerance, diet intolerance or poor-compliance will be recorded: these patients will be included in ITT analysis.

### **Sample size**

Power calculations have assumed diabetes remission in 22 % of intervention participants at one year compared with 5 % in the control group. An interclass correlation coefficient of 0.05 for weight change has been assumed to account for cluster randomisation. Allowing for an estimated 25 % individual participant drop-out within 12 months, we will recruit a total of 280 participants. Recruitment of at least 10 participants per practice for 25 practices will provide over 80 % power at alpha =0.05. Recruitment of 30–35 practices will allow for possible GP practice dropouts. In the event that lower numbers volunteer per practice, the power calculation will be revised, to re-set the recruitment plan.

## Discussion

In the UK, as in the rest of the world, the prevalence of diabetes (of which approximately 90 % is T2DM), continues to rise. This is a particularly serious problem for younger people with more extreme levels of obesity with potentially many years of future hyperglycaemia and other weight-related features of Metabolic Syndrome. In the past, T2DM was commonly referred to as ‘mild diabetes’. It is now recognised as a devastating progressive disease, which causes accelerated aging macrovascular complications affecting heart, legs and brain, plus microvascular damage to eyes, kidneys and nervous system. These sequelae have lessened to some extent with modern evidence-based treatments under clinical guidelines. However, T2DM still reduces life-expectancy significantly [31, 32] and is now a very major cause of prolonged disability, and a growing burden on healthcare services and on health budgets [33] . Remission of T2DM can be achieved by substantial weight loss following either bariatric surgery [17] or hypocaloric diet [16]. While current clinical guidelines recommend bariatric surgery for obese T2DM patients, there is little realistic prospect of this being offered to most people because of surgical and follow-up resource limitations, and personal preference to avoid surgery. The DiRECT study has been designed to establish the feasibility and durability of a primary care-based dietary intervention, which, if effective could be offered routinely as soon as possible after diagnosis of T2DM.

To assess the efficacy and safety of the DiRECT intervention, and the additional burden to patients and to staff, outcomes will be compared with those from a non-intervention control sample of similar participants with T2DM, using random allocation. Recognising the undoubted value of current clinical evidence-based guidelines, it was considered essential that both groups should continue to be managed under current UK guidelines from NICE and SIGN [11, 12], and that any new guidelines, emerging during the course of the trial, should be applied equally to all participants.

This trial is designed to be as ‘realistic’ as possible, so that the results can be assessed in the context of conventional chronic disease management, and to allow maximal transferability into routine healthcare. In the UK, most patients with T2DM are now managed in NHS primary care at least in the earlier stages of the disease before serious vascular complications have developed. The setting for DiRECT is therefore routine NHS primary care, with the intervention being delivered by existing staff following training, and then interactive support and on-the-job mentoring being provided by the experienced research team dietitians, until practitioners are confident and competent. This is the same model as evaluated in the UK feasibility study of the Counterweight-Plus weight management programme [21], which is now being offered in some health boards as part of routine NHS primary care.

A cluster randomised intervention was chosen to avoid contamination, which would be inevitable with randomisation at an individual level within GP practices, and to avoid the conflict which would arise for the dietitian or nurse if asked to offer a radically different intensive management only to randomly selected patients from their practice. The training and mentoring of practice staff delivering the programme was designed to ensure fidelity in programme delivery, necessary when interventions are delivered in more than one centre, but there always remains a possibility that differences will remain between centres. We therefore applied a conservative inter-class correlation of 0.05, which requires a 25 % increase in numbers recruited into the study. In our experience, the ICC (inter-class cluster effect) is likely to be smaller than this. In our Counterweight feasibility study, using the same intervention in 22 General Practices, there was no evidence of a cluster effect. If this applies to the present study, it is likely to be modestly over-powered.

A key principle behind the methodology of both DiRECT and the earlier Counterweight Plus feasibility study [21] is the aim to assess the impacts from maximising weight losses and long-term maintenance *for the maximum number of people*. This is subtly different from an evaluation of a specific treatment, such as a drug which is given in fixed dose. Optimising weight control entails complicated behavioural modifications in the context of busy lives and often conflicting personal knowledge and attitudes, within a wide variety of changeable social and domestic situations. It would certainly have been possible to have tested a rigid diet and lifestyle prescription, but an ‘off-the-shelf’ intervention could result in a very wide range of weight losses between individuals, with resulting wide range of secondary metabolic changes. The Counterweight feasibility study indicated that while about 25 % of patients followed the TDR fully for 12 weeks, the remainder had adhered only partially to the plan. The final outcomes of those patients varied, but many did achieve the intended 15 kg weight loss, and feedback indicated that they valued some flexibility which could accommodate individual circumstances and preferences in order to maximize weight losses. A fixed diet and lifestyle prescription cannot deliver a fixed weight-loss over a fixed time-period in a real-life setting. The decision was therefore taken that the DiRECT study would adopt a protocol which included flexibility (within pre-defined ranges) for the duration of the different phases of the intervention. Thus, it was planned that each participant should aim to lose *at least* 15 kg, (more if possible), but allow some to cease TDR early (if they were finding it difficult) and move on to FR, and others to extend TDR if there has been interruptions to protocol adherence, or they wished to continue longer. Defined windows of flexibility were incorporated into the protocol for each phase of weight-management, including pre-planned relapse-management plans for those whose weights begin to rise, or whose T2DM returned, during the maintenance phase. In a real-life, routine setting, these would be normal and expected components of care.

The aim of DiRECT is to achieve remission of diabetes, which was hypothesised would follow substantial weight loss, of the order of 15 kg. To achieve this, the duration of the weight loss intervention for DiRECT had to be at least 3 months, followed by a longer period to establish and optimise weight loss maintenance. Most clinical guidelines require evidence over 12-months or longer. The feasibility study [21] had evaluated the Counterweight-Plus programme at 12 months, but not longer. For this reason, for the power estimations, the co-primary outcome measures were 12-month changes: weight loss >15 kg and HbA1c <48 mmol/mol) analysed as binary outcomes.

The choice of HbA1c cut-off for the co-primary outcome measure was between 48 mmol/mmol (the diagnostic threshold for T2DM) and 42mmo/mmol (diagnostic threshold for IGT/pre-diabetes). There is existing evidence that weight loss of 5-10 kg (mean 4 kg at 4 years) will prevent the development of T2DM for 58 % of patients with IGT/pre-diabetes, and that ~55 % will revert to a completely normal HbA1c of <42 mmol/mmol [34]. The aim of DiRECT being to achieve remission of established T2DM, it was considered most appropriate to adopt the cut-off 48 mmol/mmol for the primary outcome measure, with 42 mmol/mmol as a secondary outcome measure.

## Conclusion

The DiRECT study, whose results will be available from 2018, aims to provide evidence to inform and guide future strategy to enhance the management of T2DM. It evaluates an existing, but not yet recommended, approach for routine weight management. The experimental protocol is patient-centered, and flexible within defined limits, in order to optimize weight loss and maintenance for the maximum number of patients. Its aims are to reverse the diagnosis of T2DM and normalise other associated metabolic features for substantial proportion of participants. With effective long-term weight loss maintenance, this should allow freedom for patients from the limitations which diabetes places on everyday life, occupation, insurance etc. In addition, it might potentially reduce morbidity from diabetic complications, but the present study is not powered for this purpose.

## Appendix 1: Protocols for management of blood pressure & inadequate glucose control

### Blood pressure

#### Background

Antihypertensive and diuretic drugs will be stopped on the day the total diet replacement is commenced. This is a safety measure, because blood pressure is likely to fall on the diet. This protocol lays out the standard approach to be followed to allow clear description of the research findings. Individual clinical decisions may be necessary for a person’s best interest. The level of 140 mmHg is chosen to allow safe decisions during the weight loss period. After the Food Reintroduction period follow usual guidelines for management of hypertension. To simplify decision making, systolic pressure only is used as a guide to therapy even though both systolic and diastolic are relevant to long term benefit.

#### Protocol

##### When antihyperensive drugs are stopped, re-emphasise the importance of avoiding sodium (salt)

1. In the first 2 weeks after stopping antihypertensives and diuretics:
  If systolic BP over 165 mmHg on repeated measurement - restart one drug, as below.
2. Thereafter, if systolic BP is >140 mmHg - restart one drug as below.
3. Increase dose weekly to achieve target.
4. If systolic BP remains >140 mmHg on the first drug - add a second drug, as below.
5. Increase dose weekly to achieve target.
6. Repeat as necessary with third, fourth or more drugs (increasing each to maximum dose).

##### Order of reintroduction of previously used drugs

1. ACE inhibitors (ramipril. lisinopril, perindropril, etc.)
2. Angiotensin receptor blockers (irbesartan, candesartan etc.)
3. Thiazide type (bendroflumethazide, indapamide etc.)
4. Spironolactone
5. Calcium channel blocker (nifedipine, amlodipine etc.)
6. Beta blocker (atenolol, labetolol etc.)
7. Alpha blocker (doxazosin, prazosin)
8. All others

### Glucose control

#### Background

All anti-diabetic drugs will be stopped on the day Total Diet Replacement (TDR) is commenced. This is a safety measure because blood glucose levels fall rapidly on the diet.

#### Protocol

1. After 2 weeks of TDR if osmotic symptoms (thirst, polyuria) are troublesome or if random capillary glucose is over 20 mmol, check HBA1c and that weight loss is as anticipated.
2. If it is not, discuss whether any other help would be helpful with following the low calorie liquid diet.
3. If weight loss is satisfactory but control is still inadequate, consider introducing an oral hypoglycaemic agent.
4. Start at the lowest dose and increase gradually.
5. Subsequently, if control remains poor, add further agents
6. Urge further weight loss at each visit

#### Order for reintroduction of anti-diabetic medications

1. Reintroduce metformin (500 mg bd). If this has previously caused GI upset for the individual, use the slow release preparation.
2. Increase metformin to a usual maximum of 1 g BD over 2–4 weeks
3. If a second agent is required, add sitagliptin 100 mg od.
4. If, after 4 weeks, control is still inadequate, add gliclazide 80 mg od (or other sulphonylurea if previously used, or if preferred).
5. Increase sulphonylurea dose gradually until glucose control is adequate
6. Use current diabetes guidelines if glucose control remains inadequate

## Additional file

Additional file 1: Table 1. DiRECT schedule of assessments. (DOCX 26.4 kb)
