# Supplementary material for: The Diabetes Remission Clinical Trial (DiRECT): protocol for a cluster randomised trial
Source: BMC Fam Pract. 2016 Feb 16;17:20. doi: 10.1186/s12875-016-0406-2 (PMC4754868; doi:10.1186/s12875-016-0406-2)
Supplement: Additional file 1: Table 1. — DiRECT schedule of assessments. (DOCX 26.4 kb) [file 12875_2016_406_MOESM1_ESM.docx]

**Table 1:** DiRECT schedule of assessments

|  | | | **Total Diet Replacement Phase** | | | | | | | | | | | | | | | | **Food Reintroduction Phase** | | | | | | |
| --- | --- | --- | --- | --- | --- | --- | --- | --- | --- | --- | --- | --- | --- | --- | --- | --- | --- | --- | --- | --- | --- | --- | --- | --- | --- |
| **Appointment** | **0** | | **1** | | | **2** | | **3** | | **4** | | | **5** | | **6** | | **7** | | **8** | | **9** | | | **10** | **11** |
| **Week** | **0 (Baseline)** | | **0+1** | | | **0+2** | | **0+4** | | **0+6** | | | **0+8** | | **0+10** | | **0+12** | | **0+13** | | **0+15** | | | **0+17** | **0+19** |
| **Study Procedure** | **Outcome Data Collection** | |  | | |  | |  | |  | | |  | |  | |  | |  | |  | | |  |  |
| Review & discuss study participation **^a^** | **√** | |  | | |  | |  | |  | | |  | |  | |  | |  | |  | | |  |  |
| Review Inclusion/Exclusion Criteria **^a^** | **√** | |  | | |  | |  | |  | | |  | |  | |  | |  | |  | | |  |  |
| Obtain Informed Consent **^a^** | **√** | |  | | |  | |  | |  | | |  | |  | |  | |  | |  | | |  |  |
| Height **^a^** | **√** | |  | | |  | |  | |  | | |  | |  | |  | |  | |  | | |  |  |
| Weight | **√** | | **√^c^** | | | **√^c^** | | **√^c^** | | **√^c^** | | | **√^c^** | | **√^c^** | | **√^c^** | | **√^c^** | | **√^c^** | | | **√^c^** | **√^c^** |
| Waist Circumference | **√** | |  | | |  | |  | |  | | |  | |  | | **√^c^** | |  | |  | | |  | **√^c^** |
| Hip Circumference | **√** | |  | | |  | |  | |  | | |  | |  | |  | |  | |  | | |  |  |
| Blood Pressure | **√** | | **√^c^** | | | **√^c^** | | **√^c^** | | **√^c^** | | | **√^c^** | | **√^c^** | | **√^c^** | | **√^c^** | | **√^c^** | | | **√^c^** | **√^c^** |
| Blood & urine samples | **√** | |  | | |  | |  | |  | | |  | |  | |  | |  | |  | | |  |  |
| Capillary blood glucose |  | | **√^c^** | | | **√^c^** | | **√^c^** | | **√^c^** | | | **√^c^** | | **√^c^** | | **√^c^** | | **√^c^** | | **√^c^** | | | **√^c^** | **√^c^** |
| Repeat HbA1c |  | |  | | |  | |  | |  | | |  | |  | |  | | **√^c^** | |  | | |  |  |
| EQ-5D-3L questionnaire | **√** | |  | | |  | |  | |  | | |  | |  | |  | |  | |  | | |  |  |
| Binge Eating Questionnaire **^a^** | **√** | |  | | |  | |  | |  | | |  | |  | |  | |  | |  | | |  |  |
| Physical Activity | **√** | |  | | |  | |  | |  | | |  | |  | |  | |  | |  | | |  |  |
| MR Scanning | **√ ^b^** | |  | | |  | |  | |  | | |  | |  | |  | | **√ ^b^** | |  | | |  |  |
| Assessment of Beta Cell Function | **√ ^b^** | |  | | |  | |  | |  | | |  | |  | |  | | **√ ^b^** | |  | | |  |  |
| Whole Body substrate Oxidation | **√ ^b^** | |  | | |  | |  | |  | | |  | |  | |  | | **√ ^b^** | |  | | |  |  |
| Qualitative Interviews |  | | **√^d^** | | |  | |  | |  | | | **√^d^** | |  | |  | |  | | **√^d^** | | |  |  |
| **Weight Maintenance Phase (Year 1)** | | | | | | | | | | | | | | | | | | | | | | | | | |
| **Appointment** | **12** | | **13** | | | **14** | | **15** | | **16** | | | **17** | | **18** | | **19** | | **20** | | | **21** | | | |
| **Week** | **0+21** | | **0+23** | | | **0+27** | | **0+31** | | **0+35** | | | **0+39** | | **0+43** | | **0+47** | | **0+51** | | | **0+52** | | | |
| **Study Procedure** |  | |  | | |  | |  | |  | | |  | |  | |  | |  | | | **Outcome Data Collection** | | | |
| Weight | **√^c^** | | **√^c^** | | | **√^c^** | | **√^c^** | | **√^c^** | | | **√^c^** | | **√^c^** | | **√^c^** | | **√^c^** | | | **√** | | | |
| Waist Circumference |  | |  | | | **√^c^** | |  | |  | | | **√^c^** | |  | |  | | **√^c^** | | | **√** | | | |
| Hip Circumference |  | |  | | |  | |  | |  | | |  | |  | |  | |  | | | **√** | | | |
| Blood Pressure | **√^c^** | | **√^c^** | | | **√^c^** | | **√^c^** | | **√^c^** | | | **√^c^** | | **√^c^** | | **√^c^** | | **√^c^** | | | **√** | | | |
| Blood & urine samples |  | |  | | |  | |  | |  | | |  | |  | |  | |  | | | **√** | | | |
| Capillary blood glucose | **√^c^** | | **√^c^** | | | **√^c^** | | **√^c^** | | **√^c^** | | | **√^c^** | | **√^c^** | | **√^c^** | | **√^c^** | | |  | | | |
| EQ-5D-3L questionnaire |  | |  | | |  | |  | |  | | |  | |  | |  | |  | | | **√** | | | |
| Physical Activity |  | |  | | |  | |  | |  | | |  | |  | |  | |  | | | **√** | | | |
| MR Scanning |  | |  | | | **√^b^** | |  | |  | | |  | |  | |  | |  | | | **√^b^** | | | |
| Assessment of Beta Cell Function |  | |  | | | **√^b^** | |  | |  | | |  | |  | |  | |  | | | **√^b^** | | | |
| Whole Body substrate Oxidation |  | |  | | | **√^b^** | |  | |  | | |  | |  | |  | |  | | | **√^b^** | | | |
| Qualitative Interviews |  | |  | | |  | |  | |  | | |  | |  | |  | |  | | | **√^d^** | | | |
| **Weight Maintenance Phase (Year 2)** | | | | | | | | | | | | | | | | | | | | | | | | | |
| **Appointment** | **22** | **23** | | **24** | **25** | | **26** | | **27** | | **28** | **29** | | **30** | | **31** | | **32** | | **33** | | | **34** | | |
| **Week** | **0+56** | **0+60** | | **0+64** | **0+68** | | **0+72** | | **0+76** | | **0+80** | **0+84** | | **0+88** | | **0+92** | | **0+96** | | **0+100** | | | **0+104** | | |
| **Study Procedure** |  |  | |  |  | |  | |  | |  |  | |  | |  | |  | |  | | | **Outcome Data Collection** | | |
| Weight | **√^c^** | **√^c^** | | **√^c^** | **√^c^** | | **√^c^** | | **√^c^** | | **√^c^** | **√^c^** | | **√^c^** | | **√^c^** | | **√^c^** | | **√^c^** | | | **√** | | |
| Waist Circumference |  |  | | **√^c^** |  | |  | | **√^c^** | |  |  | | **√^c^** | |  | |  | | **√^c^** | | | **√** | | |
| Hip Circumference |  |  | |  |  | |  | |  | |  |  | |  | |  | |  | |  | | | **√** | | |
| Blood Pressure | **√^c^** | **√^c^** | | **√^c^** | **√^c^** | | **√^c^** | | **√^c^** | | **√^c^** | **√^c^** | | **√^c^** | | **√^c^** | | **√^c^** | | **√^c^** | | | **√** | | |
| Blood & urine samples |  |  | |  |  | |  | |  | |  |  | |  | |  | |  | |  | | | **√** | | |
| Capillary blood glucose | **√^c^** | **√^c^** | | **√^c^** | **√^c^** | | **√^c^** | | **√^c^** | | **√^c^** | **√^c^** | | **√^c^** | | **√^c^** | | **√^c^** | | **√^c^** | | |  | | |
| EQ-5D-3L questionnaire |  |  | |  |  | |  | |  | |  |  | |  | |  | |  | |  | | | **√** | | |
| Physical Activity |  |  | |  |  | |  | |  | |  |  | |  | |  | |  | |  | | | **√** | | |
| MR Scanning |  |  | |  |  | |  | |  | |  |  | |  | |  | |  | |  | | | **√^c^** | | |
| Assessment of Beta Cell Function |  |  | |  |  | |  | |  | |  |  | |  | |  | |  | |  | | | **√^c^** | | |
| Whole Body substrate Oxidation |  |  | |  |  | |  | |  | |  |  | |  | |  | |  | |  | | | **√^c^** | | |
| Qualitative Interviews |  |  | |  |  | |  | |  | |  |  | |  | |  | |  | |  | | | **√^d^** | | |

**^a^** baseline visit only

^b^ Tyneside participants only (intervention & control)

^c^ Intervention participants only

^d^ Random sample 20 intervention + 20 control participants
